# A Retrospective Comparative Study of Sodium Fluoride (NaF-18)-PET/CT and Fluorocholine (F-18-CH) PET/CT in the Evaluation of Skeletal Metastases in Metastatic Prostate Cancer Using a Volumetric 3-D Radiomics Analysis

**DOI:** 10.3390/diagnostics11010017

**Published:** 2020-12-24

**Authors:** Kalevi Kairemo, S. Cheenu Kappadath, Timo Joensuu, Homer A. Macapinlac

**Affiliations:** 1 Department of Theragnostics, Docrates Cancer Center, 00180 Helsinki, Finland; 2 Department of Nuclear Medicine, MD Anderson Cancer Center, Houston, TX 77030, USA; hmacapinlac@mdanderson.org; 3 Department of Imaging Physics, MD Anderson Cancer Center, Houston, TX 77030, USA; skappadath@mdanderson.org; 4 Department of Medical Oncology and Radiotherapy, Docrates Cancer Center, 00180 Helsinki, Finland; timo.joensuu@docrates.com

**Keywords:** fluorocholine, sodium fluoride, positron emission tomography, prostate cancer, bone metastases, radiomics

## Abstract

Bone metastases are common in prostate cancer (PCa). Fluorocholine-18 (FCH) and sodium fluoride-18 (NaF) have been used to assess PCa associated skeletal disease in thousands of patients by demonstrating different mechanism of uptake-cell membrane (lipid) synthesis and bone mineralization. Here, this difference is characterized quantitatively in detail. Our study cohort consisted of 12 patients with advanced disease (> 5 lesions) (M) and of five PCa patients with no skeletal disease (N). They had routine PET/CT with FCH and NaF on consecutive days. Skeletal regions in CT were used to co-register the two PET/CT scans. Bone 3-D volume of interest (VOI) was defined on the CT of PET with a threshold of HU > 150, and sclerotic/dense bone as HU > 600, respectively. Additional VOIs were defined on PET uptake with the threshold values on both FCH (SUV > 3.5) and NaF (SUV > 10). The pathologic skeletal volumes for each technique (CT, HU > 600), NaF (SUV > 10) and FCH (SUV > 3.5) were developed and analyzed. The skeletal VOIs varied from 5.03 L to 7.31 L, whereas sclerotic bone VOIs were from 0.88 L to 2.99 L. Total choline kinase (cell membrane synthesis) activity for FCH (TCA) varied from 0.008 to 4.85 [kg] in M group and from 0.0006 to 0.085 [kg] in N group. Total accelerated osteoblastic (bone demineralization) activity for NaF (TBA varied from 0.25 to 13.6 [kg] in M group and varied from 0.000 to 1.09 [kg] in N group. The sclerotic bone volume represented only 1.86 ± 1.71% of the pathologic FCH volume and 4.07 ± 3.21% of the pathologic NaF volume in M group, and only 0.08 ± 0.09% and 0.18 ± 0.19% in N group, respectively. Our results suggest that CT alone cannot be used for the assessment of the extent of active metastatic skeletal disease in PCa. NaF and FCH give complementary information about the activity of the skeletal disease, improving diagnosis and disease staging.

## 1. Introduction

Prostate cancer (PCa) is the second most common male cancer and bone is the most frequent metastatic site. Approximately 10–30% of men with prostate cancer will present with incurable advanced or metastatic disease. Patients who develop metastatic disease are commonly treated with luteinizing hormone-releasing hormone agonist/antagonist therapy, which frequently leads to tumor regression [1,2]. Unfortunately, these regressions are typically transient, with an eventual tumor regrowth as castration-resistant disease that is invariably fatal [3]. Median survival in patients who develop metastatic castration-resistant prostate cancer (mCRPC) in the modern era is approximately two years [4]. One is the most frequent metastatic site in prostate cancer, with approximately 90% of patients with mCRPC having radiological evidence of bone metastases [5]. Unlike deaths from many other types of cancer, deaths from prostate cancer are usually due to bone disease and its complications.

Fluorocholine is a common radiotracer, used especially in Europe. A high progression in cancer growth demands increased lipid synthesis for the formation of new cellular membranes: this stimulates choline transporter and the cellular uptake of choline. In the cells, choline is transformed into phosphotidylcholine (lecithin) [6]. Thus, the uptake of choline is reflecting cellular proliferation. Our systematic review in more than 3000 patients demonstrated that choline PET/CT in prostate cancer had the most clinical effect in patients with PSA recurrence [6].

Sodium fluoride-18 (NaF) is a common radiotracer as well. The Society for Nuclear Medicine and Molecular Imaging has published guidelines for ^18^F-NaF PET/CT in the US [7]. ^18^F- (fluoride ion) is exchanged for OH- (hydroxide ion), meaning that the hydroxyapatite bone matrix is transformed into fluoroapatite. High uptake of ^18^F-NaF reflects bone reactions to skeletal disease, not necessarily reactions to cancer. Therefore, positive findings with ^18^F-NaF PET/CT may be due to both benign and malignant bone disorders. The uptake of ^18^F-NaF is part of the mineralization of bone matrix, and thus bone uptake of ^18^F-NaF reflects bone remodeling. Patients with positive ^18^F-NaF PET/CT scans have a high risk to develop bone metastases. In a systematic review, ^18^F-NaF PET/CT 1289 (33%) patients out of 3918 patients had positive scans [8].

It is important to characterize active skeletal metastatic disease in prostate cancer. Fluorocholine-18 (FCH) and sodium fluoride-18 (NaF) are registered radiopharmaceuticals in more than 15 EU countries that have been used to assess PCa and associated bone metastases in several thousands of patients. The aim of this study was to analyze the role of FCH-PET, NaF-PET and CT simultaneously in advanced metastatic skeletal disease.

## 2. Materials and Methods

### 2.1. Patients

Prostate cancers were diagnosed between 1999 and 2015. In this retrospective one institute analysis we included all those patients who had both [^18^F]-NaF-PET and [^18^F]-fluorocholine-PET as a part of clinical staging on consecutive days as a part of their diagnostic program.

#### 2.1.1. M Group

Twelve new consecutive patients were analyzed between August 2015–December 2015 without any further selection. Men’s ages ranged from 57 to 86 years; Gleason scores were 6–10; initial PSA values varied from 9.8 to 790 μg/L, and all patients had T3-T4 disease. In addition, all patients had skeletal metastases, and three of them had visceral metastases. At Docrates Cancer Center in Helsinki, the radiological TNM staging was performed using [^18^F]-NaF-PET and [^18^F]-fluorocholine-PET in all patients; most (10 out of 12) of the patients also had pelvic MRI and diagnostic whole body CT. These patients had to have more than five identifiable active metastases on both PET studies (in order to confirm skeletal metastatic disease).

Three of them had previous surgery (2 RRP+1TURP), seven had previous radiotherapy, all 12 had androgen deprivation therapy (ADT), six patients had received chemotherapy, three had received Sm-153-EDTMP therapy and two Ra-223, and eight additionally received denosumab. The patient characteristics are summarized in Table 1 (Arabic numbers).

#### 2.1.2. N Group

Five patients with prostate cancer who had no skeletal metastases served as controls and their characteristics are summarized in Table 1 (Roman numbers). These were the five newest patients studied at the same time frame (the study order is shown in Table 1). Their radiological TNM staging or re-staging was performed using [^18^F]-NaF-PET and [^18^F]-fluorocholine-PET on consecutive days. The men’s ages ranged from 50 to 93 years; Gleason scores were 6–9; initial PSA values varied from 7.1 to 73 μg/L, and these patients had T1c-T4 disease.

### 2.2. Imaging PET/CT Protocol

All the patients were studied for fluorocholine ^18^F-FCH-PET/CT at Docrates Cancer Center using TruePoint PET-CT (Siemens Biograph 6, Erlangen, Germany). Patients were injected with ^18^F-FCH, using an average activity of 280 MBq (range 210–402 MBq). Imaging was done in two phases, early whole body imaging at an average time of 11 ± 4 min post injection, and late imaging of the pelvis at an average time of 64 ± 5 min post injection. Whole body imaging covered the area from the calvarium to the mid-thighs.

For sodium fluoride (Na^18^F)-PET/CT imaging was performed at 62 min (range 58–76 min) as a whole body imaging (from the calvarium to the tips of toes) with an average activity of 222 MBq (range 192 to 251 MBq).

Image data sets are FCH PET/CT with nominal administered activity 280 MBq, uptake time 60 min, and scan range apex to mid-thigh. Image data sets also include NaF PET/CT with nominal administered activity 220 MBq, uptake time 60 min, and whole body scan range. Both PET/CT scans were performed on the same PET/CT scanner within 1 day of each other. 17 patients were analyzed in this work—12 patients with skeletal metastases and 5 patients with no metastases.

Both tracers were used under special permission from the Finnish Medical Evaluation Agency (FiMEA). The tracers were manufactured and supplied by MAP Medical Technologies Oy, Tikkakoski, Finland.

### 2.3. Image Aanalysis

Image segmentation for evaluation of functional volumes were performed using MIM Maestro v6.5 (MIM Software Inc., Cleveland, OH, USA). The image analysis processing steps were as follows:

1. The skeleton VOI was segmented by selecting voxels with HU > 150 on the CT scans from FCH and NaF PET/CT scans
2. A rigid registration of the two skeleton VOIs from FCH PET/CT and NaF PET/CT scans was performed. Now the two PET and CT scans are spatially co-registered in a single frame of reference. The skeleton VOI was labeled as “Skeleton” for figures.
3. A subset VOI of sclerotic (pathologic) bone was created by selecting voxels with HU > 600 within the skeleton VOI—labeled as “Skeleton 600”.
4. A PET based VOI labeled “FCH PET 3.5” was created that is the fusion of voxels with FCH PET SUVbw > 3.5 and the “Skeleton” VOI.
5. A PET based VOI labeled “NaF PET 10” was created that is the fusion of voxels with NaF PET SUVbw > 10 and the “Skeleton” VOI.
6. A PET based VOI labeled “NaF 10 FCH 3.5” was the fusion of “FCH PET 3.5” VOI and “NaF PET 10” VOI.
7. A PET based VOI labeled “Scl FCH 3.5” was the fusion of “FCH PET 3.5” VOI and “Skeleton 600” VOI.
8. A PET based VOI labeled “Scl NaF 10” was the fusion of “NaF PET 10” VOI and “Skeleton 600” VOI.
9. A PET based VOI labeled “Scl NaF 10 FCH 3.5” was the fusion of “Scl FCH 3.5” VOI and “Scl NaF 10” VOI.

In summary, we created eight VOIs for each patient based on spatially co-registered FCH PET/CT and NaF PET/CT scans; namely, “Skeleton”, “Skeleton 600”, “FCH PET 3.5”, “NaF PET 10”, “NaF 10 FCH 3.5”, “Scl FCH 3.5”, “Scl NaF 10”, and “Scl NaF 10 FCH 3.5”. For each VOI we computed the VOI volume (in ml). The thresholds for skeleton (HU > 150) and dense bone (sclerosis) (HU > 600) were determined from the CT component of the PET/CT study and were based on experience in more than 500 PET/CT studies. The pathologic FCH value (SUV > 3.5) was based on experience in more than 300 FCH/PET studies to indicate a pathological uptake. Tha pathological NaF value (SUV > 10) was similarly based on individual experience, but it was the same as the MDACC TFL_10_ criteria presented in the literature [9].

**Figure 1:**
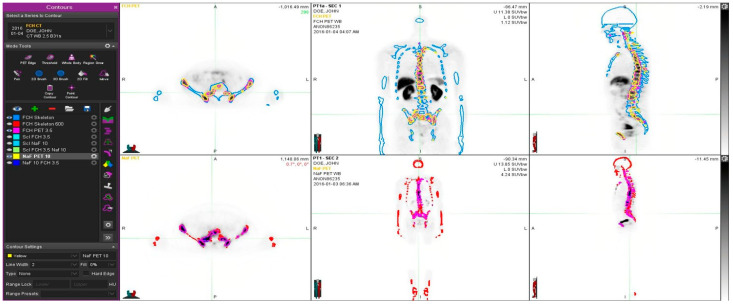
Example of a patient study (pt 11, Table 1). This 67-year old patient had an aggressive prostate cancer, T4N1M1 when initially diagnosed with Gleason score 9. S-PSA was 40. The upper row shows FCH PET study one transaxial, coronal and sagittal section. The lower row illustrates the same sections in the NaF PET study. Here, normal skeleton (HU > 150 as measured from simultaneous CT) is shown in red on NaF PET (lower row) and blue on FCH PET (upper row). Pathologic NaF (SUV > 10) is shown in yellow on FCH PET (upper row) and pathologic FCH (SUV > 3.5) in purple on NaF PET (lower row). This demonstrates clearly that volume regions differ significantly from each other, and PET tracers show different distributions, i.e., NaF is targeting cortical bone and FCH bone marrow.

In addition to the PET based VOIs, we computed metrics analogous to total lesion glycolysis (TLG) in FDG PET scans. For the FCH PET based VOIs, the product of the VOI volume and the mean SUVbw in “FCH PET 3.5” and “Scl FCH 3.5” corresponds to the total cell membrane synthesis activity (TCA) in bone and sclerotic bone, respectively. For the NaF PET based VOIs, the product of the VOI volume and the mean SUVbw in “NaF PET 10” and “Scl NaF 10” corresponds to the total bone demineralization (TBA) in bone and sclerotic bone, respectively. An example of the patient analysis is shown in Figure 1.

The percentage volumes of “Skeleton 600”, “FCH PET 3.5”, “NaF PET 10”, and “NaF 10 FCH 3.5” with respect to “Skeleton” were calculated. Similarly, the percentage volumes of “Scl FCH 3.5”, “Scl NaF 10”, and “Scl NaF 10 FCH 3.5” with respect to “Skeleton 600” was calculated. The mean, minimum, and maximum of the percentage volumes were calculated. The ratio of mean percentage volumes between abnormal and normal patients were calculated. A student’s t-test was performed between the percentage volumes for abnormal and normal patients.

## 3. Results

Our cohort consisted of twelve patients with advanced disease (> 5 lesions) who had had routine PET/CT both with FCH and NaF on consecutive days. An example is given in Figure 1.

The patient characteristics are summarized in Table 1. For comparison, we had five patients diagnosed with prostate cancer who did not have skeletal disease (Table 1).

Our results demonstrate that the skeletal VOI volumes varied from 5.03 L to 7.31 L in patients with skeletal metastases (*n* = 12), whereas sclerotic bone volumes were from 0.88 L to 2.99 L. The sclerotic bone volume was 20.5 ± 6.8% of skeletal volume (this was defined by FCH-PET).

In control prostate cancer patients without skeletal metastases, the skeletal VOI volumes varied from 4.60 L to 7.90 L (*n* = 5), and sclerotic bone volumes were from 1.02 L to 1.76 L. The sclerotic bone volume was 19.9 ± 4.4% of skeletal volume.

The TCA varied from 0.008 to 4.85 [kg] in patients with skeletal metastases and varied from 0.0006 to 0.085 [kg] in PCa control patients with no metastases. The TBA varied from 0.25 to 13.6 [kg] in patients with skeletal metastases and varied from 0.000 to 1.09 [kg] in PCa control patients.

The sclerotic bone volume represented only 1.86 ± 1.71% of the pathologic FCH volume and 4.07 ± 3.21% of the TLF_10_ (SUVmax >10) in patients with multiple metastases. In the control PCa, patients pathologic FCH was only 0.08 ± 0.09% of the sclerotic bone volume (*p* < 0.004) and pathologic NaF volume 0.18 ± 0.19% of sclerotic bone (*p* < 0.001). All the differences between the metastasis group and control cancer patient group are shown in Table 2.

Table 2 summarizes pathologic PET/CT volumes as percentages of the skeletal volumes (when HU > 150 on CT). Pathologic volumes for FCH (SUV >3.5), NaF (SUV > 10) and when both were pathologic on PET were larger in patients with metastases as compared to patients with no metastases. Sclerosis as measured from CT (HU >600) and this in combination with pathologic FCH (SUV >3.5) and NaF concentrations (SUV > 10) were similarly larger in patients with metastases. All the differences were statistically significant (Table 2).

**Figure 2:**
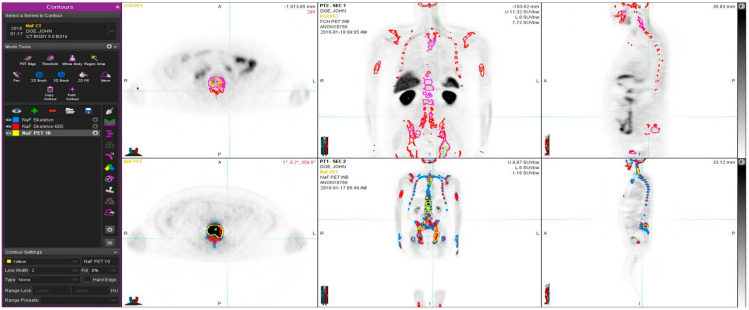
Example of a patient study (pt 12, Table 1). This 50-year old patient had an aggressive prostate cancer, T4N1M1 with initial PSA 700 and Gleason score 9. He was treated with radiation therapy, hormonal therapy, chemotherapy and multiple targeted bone therapies. At the time of the imagings S-PSA was 90. The upper row shows FCH PET study one transaxial, coronal and sagittal section. The lower row illustrates the same sections in the NaF PET study. Here, normal skeleton (HU > 150, as measured from simultaneous CT), is shown in blue on NaF PET (lower row). Sclerotic bone (HU > 600) is shown in red in all images. Pathologic NaF (SUV > 10) is shown in yellow (lower row) on NaF PET and in purple on FCH PET (upper row). This demonstrates clearly that volume regions differ significantly from each other, and PET tracers show different distributions.

Figure 2 demonstrates a young prostate cancer patient (50-year-old) with aggressive prostate cancer, and T4N1M1 with an initial PSA 700 and a Gleason score 9. He was treated with radiation therapy, hormonal therapy, chemotherapy and multiple targeted bone therapies, including two radionuclide therapies. In spite of this, the PET volume regions differed significantly from each other, and PET tracers show different distributions. Fluoride typically targets cortical bone and bone formation, whereas choline targets active proliferative cancer cells, preferably bone marrow. The tiny overlapping regions can be seen in Figure 2, especially in the transaxial image.

**Figure 3:**
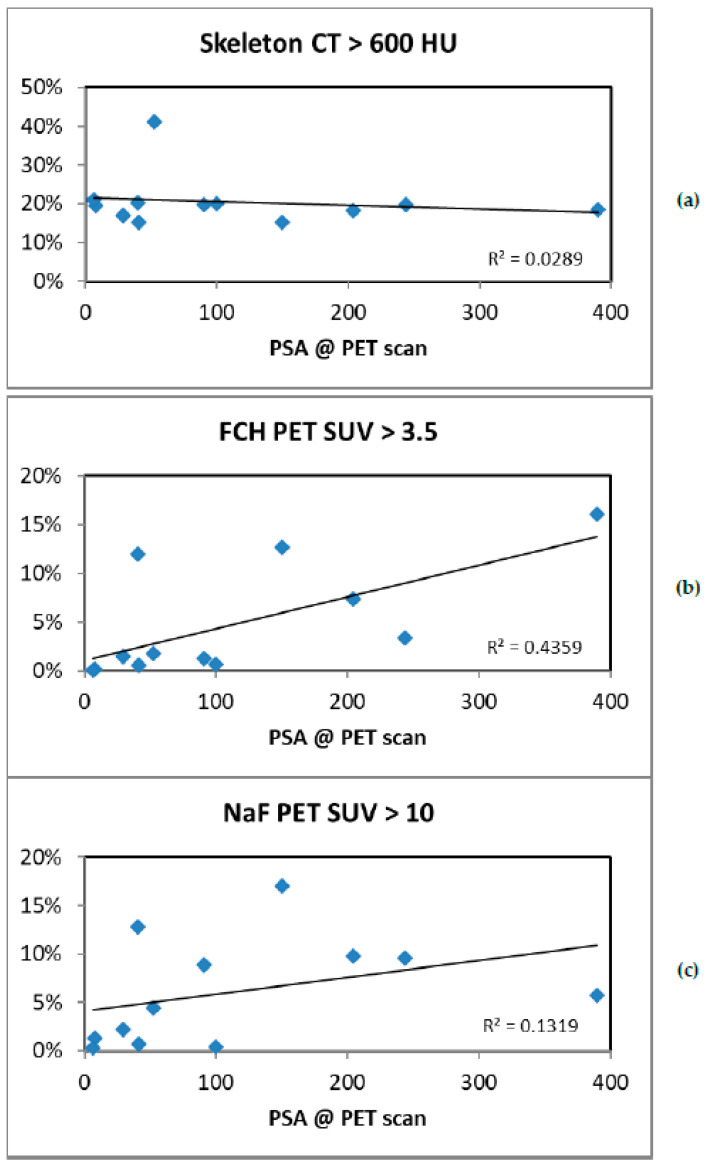
Volumes of sclerosis (HU > 600) (**a**), total choline kinase activity (SUV > 3.5 on FCH PET) (**b**) and total bone demineralization activity (SUV > 10 on NaF PET) vs. serum PSA (**c**). PSA correlated with total choline kinase activity (*p* < 0.02). PSA did not correlate with the amount of sclerosis seen on CT (*p* > 0.5) nor with the bone demineralization activity (*p* > 0.2). S-PSA is presented as ng/mL and volumes as percentages of total skeletal volume.

There was a correlation between TCA and S-PSA (*p* < 0.020), but TBA and total sclerotic bone did not correlate with S-PSA. Figure 3a–c show the correlations.

## 4. Discussion

In this study we analyzed of FCH-PET, NaF-PET and CT at the same time in advanced metastatic skeletal disease. NaF-PET/CT was performed in one session and FCH/PET-CT on the following day, the PET studies were then fixed with each other with the use of the skeletal CT. Due to this, the skeleton will not change substantially in one day, and this allows for the overlap of FCH-PET and NaF-PET three-dimensionally and the possibility of analyzing the regional differences in detail.

There was no difference in the amount of sclerosis in our patient groups, the sclerotic bone volume was 20.5 ± 6.77% of skeletal volume, as defined by FCH-PET in patients with bone metastases (M), and 19.9 ± 4.43% of skeletal volume in control prostate cancer patients without skeletal metastases (N). The sclerotic bone volume was defined from the CT measurements using Hounsfield Unit criteria, HU > 600. This indicates that the patient populations could be compared with each other for other skeletal phenomena because the degenerative changes were in the same level in both patient populations. The sclerotic volume ranges were from 0.88 L to 2.99 L in the metastatic group and from 1.02 L to 1.76 L in control patients without bone metastases.

**Figure 4:**
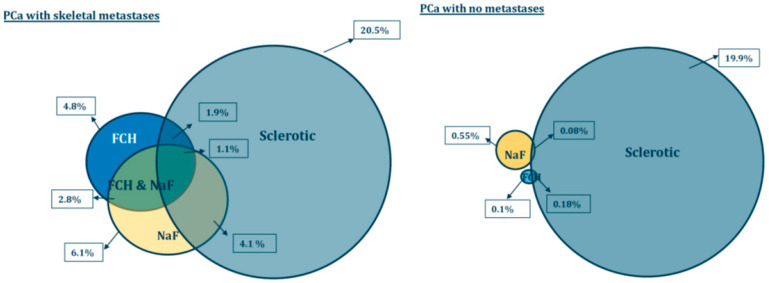
Pathologic PET/CT volumes as percentages of the skeletal volumes (when HU > 150 on CT) are presented as spheres. Pathologic volumes for FCH (SUV > 3.5) are in blue and NaF (SUV > 10) in yellow. Sclerosis is measured from CT (HU > 600) volume (grey). The volumes are shown for patients with bone metastases (M) and for control prostate cancer patients with no skeletal metastases (N). The amount of sclerosis (grey spheres) does not statistically differ in these patient groups. All pathological volumes, including overlapping regions, differ statistically significantly from each other between M and N group.

We developed new parameters (analogous to TLG, total lesion glycolysis for ^18^FDG-PET) as total choline kinase activity (total cell membrane synthesis activity) for FCH (TCA) and total accelerated osteoblastic activity (total bone demineralization) activity for NaF (TBA). The respective volumes of the whole skeletal volume are shown as Venn-Diagrams in Figure 4.

The differences are seen easily from the Figure 4. The pathologic PET variables, i.e., FCH (blue) and NaF (yellow) volumes differ significantly from each other, eventhough there is an overlapping region (2.8%) in patients with skeletal metastases. In control prostate cancer patients (with no skeletal metastases), these regions did not overlap. In clinical practice, this means that these methods measure different phenomena.

The diagnostic accuracy of ^18^F-Choline PET/CT is improved by use of both early and late imaging [10]. An article analyzing 300 patients found that 48 (16%) had false-positive results [11]. False positive findings were listed: in the prostate may be related to prostatitis and intraepithelial neoplasia; in lymph nodes to lymphadenitis; and in distant organs to other neoplasms. Evangelista et al. [12] found a sensitivity of 49% and a specificity of 95% in a systematic review. Androgen deprivation therapy (ADT) may reduce choline uptake in PET scans [13,14], but some studies did not find a reduction associated with ADT [14,15]. Choline PET/CT-positive patients had a poorer survival than PET/CT-negative patients, indicating that it is a prognostic factor [16,17,18]. In a systematic analysis, of 938 patients, 381 (41%) had positive radiolabeled choline PET/CT scans and a change of treatment, and after the change of treatment, 101 (25%) of 404 patients had a complete PSA response [6]. In a study consisting of 58 patients, it was stated that FCH PET/CT should be preferred to CT and bone scintigraphy in patients with prostate cancer with bone metastases, because it allows for a better stratification of time to progression, skeletal event free survival and cancer specific survival [19].

Our own experience with FCH PET false-positive findings associate with myeloproliferative medication in these patients with advanced and aggressive metastatic prostate cancer, such as GM-CSF treatment. In the literature, a case is presented about FCH PET/CT in a patient with biochemical recurrent prostate cancer who was receiving erythropoietin for hemochromatosis. Only diffuse skeletal uptake of FCH was seen. ^18^F-Fluoride PET/CT performed the following day demonstrated multiple abnormal focal bone metastases [20]. This is the way we analyzed all these patients: two PET studies on consecutive days. Theoretically, widespread diffuse metastazing configuration could be missed, if the judgement would be based on a single PET study. Sometimes PCa can be PSA negative, and negative PET findings occur with both NaF and FCH [21,22,23]. Two PET studies were used routinely in the diagnosis of prostate cancer in our institution. Earlier it was NaF and FCH on consecutive days, because they gave complementary information. In Figure 4, the intersection of NaF and FCH (green area) represents the same volume; what is seen either in blue or yellow is complementary information. Today, it could also be PSMA-PET and FDG-PET in order to identify those patients who do not necessarily respond to targeted therapies, such as PSMA targeted therapies [24]. With this NaF and FCH approach, we could select patients who benefit from bone targeting therapies, such as Ra-223 and select patients who might benefit from antiproliferative chemotherapy.

The data from US National Oncologic PET Registry demonstrates that the indication for the PET/CT reflects PET/CT findings: 14% of patients had a positive ^18^F-NaF PET/CT at staging; 29% of the patients had positive ^18^F-NaF PET/CT if the examination was an initial test for bone metastases; 76% of the patients had positive ^18^F-NaF PET/CT if the examination was requested as a test for progressive bone metastases [8]. ^18^F-NaF and ^18^F-Choline PET/CT show similar diagnostic accuracy at staging for patients with prostate cancer, but ^18^F-Choline has higher specificity at restaging for recurrence [21]. Sodium fluoride suits for follow-up of PCa with skeletal metastases [25,26,27] and even for quantitative assessment of treatment response [28]. In fact, quantitative criteria have been developed [29].

This dual tracer method improves diagnostics. Bone metastases are formed by hematogenic spread; therefore, the assessment of bone marrow and cortical bone separately is a clear benefit. This radiomics evaluation utilizing rigid skeleton to fix two PET studies is a new method that makes volumetric evaluations possible. Theoretically, it enables a new possibility for the detection of bone marrow/cortex interface activity. In spite of the limited number of patients, 17 total, we could find in almost every patient small areas of FCH and NaF concentrations, which did not overlap. These should be characterized in detail in larger populations because bone sampling is impossible. Even though our findings are very statistically significant, the small number of patients may have an effect on results. Nevertheless, we have shown here identical results in a patient who was treatment naively (Figure 1) and in a patient who was heavily treated, including targeted bone therapies (Figure 2); in both patients with T_4_N_1_M_1_ disease, the methods gave similar complementary information. There are multiple options for PET tracer selection in prostate cancer [30]. This dual tracer combination for volumetric radiomics analysis seems to be excellent, but other alternatives should be tested in clinical trials; PSMA is an important new target.

## 5. Conclusions

From this study, we can conclude that CT is not well suited for assessing active skeletal metastases in an aggressive prostate cancer (T3-4 disease). Both NaF and FCH are useful in the evaluation of active skeletal metastatic disease, but they measure different phenomena and definitely give complementary information. Furthermore, FCH correlates positively with serum PSA concentration and is thus probably a prognostic factor.

## Figures and Tables

**Table 1:** Patient characteristics. Age, Gleason score (GS), TNM classification, time of diagnosis, initial S-PSA, previous treatments, skeletal therapies and S-PSA at the time of analysis. Abbreviations: ADT—androgen deprivation, Ch—chemotherapy, Rx—radiotherapy, A—abiraterone, D—denosumab, Ra—radium-223, Sm—Sm-153, Z—zolendronate. Arabic numbers refer to patients with skeletal metastases (M) and Roman numbers to patients with no skeletal disease (N).

No.	Age	GS	TNM	Dgn	iPSA	Previous Treatments	Bone Therapies	PSA at PET-CT
**1**	86	6	T1cNxMx	I–04	9.8	TURP, ADT		204
**2**	76	8	T3NxM0		400	Rx, ADT	A, Z	41
**3**	74	8	T3N0M0	X–11	126	Rx, ADT, Ch	Z, Sm, D	6.56
**4**	57	10	T3N0M1	VII–13	790	Rx, ADT, Ch	Rx, D, Sm, Ra	52.4
**5**	85	9	T3bN1M1	IX–15	100	Rx, ADT	Rx, D	100
**6**	63	8	T3NxM1	IV–13	490	ADT, Ch	D	29
**7**	69	9 (4 + 5)	T3bN1M1	III–14	86.4	ADT, Ch	Rx, D	7.83
**8**	59	7 (3 + 4)	T3bN0M0	VI–10	13	RRP, ADT, Ch	D	150
**9**	76	7 (3 + 4)	T2bN0M0	IV–02	16	RRP, ADT, Ch	D	390
**I**	61	7 (3 + 4)	T1cN0M0	XII–15	7.1			8
**10**	61	8	T3aN0M1	XI–15	244	ADT		244
**11**	67	9 (4 + 5)	T4N1M1	XII–15	40			40
**II**	70	7 (4 + 3)	T3bN0M0	XII–15	17.6			17.6
**III**	70	6	T3bN0M0	XII–15	12.6			12.6
**IV**	93	5	T1cN0M0	III–99	10.5	ADT		26
**V**	76	7 (4 + 3)	T4N0M0	XII–15	73			73
**12**	50	9	T4N1M1	II–13	700	Rx, ADT, Ch	Rx, D, Sm, Ra	90.5

**Table 2:** Results. Pathologic PET/CT volumes as percentages of the skeletal volumes (when HU > 150 on CT) are presented. Pathologic volumes for FCH (SUV > 3.5), NaF (SUV > 10) and when both are pathologic on PET studies are shown. Sclerosis is measured from CT (HU > 600) volume and calculated from FCH PET/CT. Similarly, simultaneous sclerosis and pathologic volumes for both FCH and/or NaF are shown. The volumes are shown for patients with bone metastases (M) and for control prostate cancer patients with no skeletal metastases (N). The amount of sclerosis does not statistically differ in these patient groups. All pathological volumes differ statistically significantly from each other between M and N group.

	FCH-PET (SUV > 3.5)	NaF-PET (SUV > 10)	FCH- & NaF-PET (SUV > 3.5 & > 10)	Sclerosis on CT (HU > 600)	Sclerosis & FCH (HU > 600 & SUV > 3.5)	Sclerosis & NaF (HU > 600 & SUV > 10)	Sclerosis & FCH & NaF (HU > 600 & SUV > 3.5 & > 10)
Skeletal mets (*n* = 12)	4.8 ± 5.7%	6.1 ± 5.5%	2.8 ± 3.2%	20.5 ± 6.8%	1.9 ± 1.7%	4.1 ± 3.2%	1.1 ± 1.0%
PCa with no mets (*n* = 5)	0.11 ± 0.11%	0.55 ± 0.57%	0.00 ± 0.00%	19.9 ± 4.3%	0.08 ± 0.09%	0.18 ± 0.19%	0.00 ± 0.00%
*p*-value	<0.017	<0.005	<0.013	0.843	<0.004	<0.001	<0.002
